# Comparison of Gut Microbiota between Sasang Constitutions

**DOI:** 10.1155/2013/171643

**Published:** 2013-12-25

**Authors:** Bong-Soo Kim, Hyo Sang Bae, Chi-yeon Lim, Mi Jeong Kim, Jae-gu Seo, Jong Yeol Kim, Jai-eun Kim, Hojun Kim

**Affiliations:** ^1^ChunLab, Inc., Seoul National University, Seoul 151-742, Republic of Korea; ^2^Department of Sasang Constitutional Medicine, Dongguk University, 814 Siksa-dong, Gyeonggi-do 410-773, Republic of Korea; ^3^Department of Medicine, Dongguk University, 814 Siksa-dong, Gyeonggi-do 410-773, Republic of Korea; ^4^Department of Food and Nutrition, Silla University, Busan 617-736, Republic of Korea; ^5^Cell Biotech Co., Ltd., Gimpo 415-871, Republic of Korea; ^6^Korea Institute of Oriental Medicine (KIOM), Daejeon 305-811, Republic of Korea; ^7^Department of Pathology, College of Oriental Medicine, Dongguk University, Seoul 410-773, Republic of Korea; ^8^Department of Rehabilitation Medicine of Korean Medicine, Dongguk University, 814 Siksa-dong, Gyeonggi-do 410-773, Republic of Korea

## Abstract

The Sasang constitutional medicine has long been applied to diagnose and treat patients with various diseases. Studies have been conducted for establishment of scientific evidence supporting Sasang Constitutional (SC) diagnosis. Recent human microbiome studies have demonstrated individual variations of gut microbiota which can be dependent on lifestyle and health conditions. We hypothesized that gut microbial similarities and discrepancies may exist across SC types. We compared the difference of gut microbiota among three constitutions (So-Yang, So-Eum, and Tae-Eum), along with the investigation of anthropometric and biochemical parameters. Firmicutes and Bacteroidetes were predominant phyla in all SC types. The median plot analysis suggested that Firmicutes and Bacteroidetes appeared more abundant in SE and TE, respectively, in the male subjects of 20–29 years old. At the genus level, *Bifidobacterium* and *Bacteroides* manifested the difference between SE and TE types. For anthropometry, body weight, body mass index, and waist circumference of the TE type were significantly higher than those of the other types. Overall, findings indicated a possible link between SC types and gut microbiota within a narrow age range. Further investigations are deemed necessary to elucidate the influences of age, gender, and other factors in the context of SC types and gut microbiota.

## 1. Introduction

Individualized treatment according to the patients' “syndrome” pattern is one of the characteristics of traditional oriental medicine in the East Asian countries. The Sasang constitutional medicine, a holistic approach based on the unique constitutional typology, has long been applied to diagnose and treat patient with various diseases. Characteristics pertaining one's physical, psychosomatic, and emotional aspects are integrated in the determination of Sasang constitutional (SC) types. The SCM deals with biological and psychological traits in individuals and explains individual differences in behavioral tendencies, physical characteristics, and varying levels of vulnerability to disease and responsiveness to environmental stimuli. Diagnosis under the SC typology divides human beings into four categories (Tae-Yang, Tae-Eum, So-Yang, and So-Eum) according to their inherited traits, including appearance, physiology, susceptibility to disease, and personality. Traditional Korean medical doctors classify patients using SC for diagnosis and treatment in clinics. However, like other forms of complementary and alternative medical systems worldwide, the identification of SC has been subjective and fallible because of lack of standardization and scientific evidence for a long time [1]. Therefore, with development of new biomedical technologies, various attempts at scientific identification of SC have been made. Several studies have reported that a specific genetic polymorphism is related to a specific constitution [2, 3] and each SC has a unique gene expression pattern [4–6]. Specific constitutions are also found to have significant epidemiological relationships with some diseases or symptoms [7–10]. These studies can aid in accurate diagnosis and treatments using identification of SC by establishment and accumulation of scientific measurements.

Recent advances in sequencing technology have provided extended information on the microbiome in the bodies of healthy individuals [11, 12]. Among body sites, the highest number of bacterial cells was observed (up to 10^12^ cells per gram) in the intestine, and the diversity of microbes was higher in the intestine, compared with other sites [12, 13]. Gut microbiota play beneficial roles in human health, including immunity, nutrition, and protection from pathogens [14]. Several studies have reported an association of disruptions of normal gut microbiota with metabolic disease [15–17]. Individual variations of indigenous gut microbiota have been reported in several studies and gut microbiota were influenced by various factors [12, 18–20]. These variations and metabolic interaction of gut microbiota with host could be related to identification of SC. In this study, we investigated and compared gut microbiota between different SC using pyrosequencing based on the 16S rRNA gene. In addition, the comparison of metabolic markers among the different constitutions was also investigated. The results of this study could be used as one of the factors in determination of SC in traditional Korean medicine.

## 2. Materials and Methods

### 2.1. Study Subjects

Subjects were recruited to Dongguk University Ilsan Hospital by advertisements in the local newspaper or by posters in the hospital with approval of the Institutional Review Board of Dongguk University Ilsan Hospital (approval no. 2012-SR-27). For qualification, subjects were normal weight (BMI < 25 kg/m^2^), male or female, between the ages of 19 and 65. They had been weight-stable within ±10% during the last six months. Any antibiotics, probiotics, or drugs that might have an impact on their weight were not used for the last three months. Subjects with weight-influencing diseases, including hyper/hypothyroidism, heart disease, psychogenic disease, or other chronic systemic disease, were excluded. Smokers or pregnant women confirmed by positive screening on the hCG test were also excluded. A total of 40 subjects were recruited (14 Tae-Eum, 13 So-Yang, and 13 So-Eum) for this study. Subjects underwent physical examinations, body composition analysis, anthropometry, blood test, and Sasang constitutional diagnosis.

### 2.2. General Characteristics of Subjects

Blood pressure and heart rate were measured using an automatic digital sphygmomanometer. Body weight and height were measured using an automatic scale (G-tech, Uijeongbu, Korea), wearing a hospital gown (the nearest 0.1 kg and 0.5 cm, resp.). Body mass index was calculated by weight in kilograms and divided by height in meters squared. Waist circumference was measured three times according to the WHO instruction [21]. Body composition was measured using the bioelectrical impedance analysis method (InBody 3.0, Biospace, Seoul, Korea). This device measures impedance through eight tactile electrodes placed on palms, thumbs, heels, and soles. Each subject stood upright stepping on the foot electrodes and loosely gripping the pipe-shaped hand electrodes with arms held vertically. Values for lean body mass, fat mass, fat percentage, and waist-to-hip ratio were obtained using the device mentioned above. Blood tests, including fasting glucose, HDL-cholesterol, triglyceride, total cholesterol, and AST/ALT, were performed using the Cobas 8000 modular analyzer (Roche, Branford, CT, USA).

### 2.3. Statistical Analyses

Continuous data are expressed as mean ± standard deviation (SD) and categorical data were described by frequency. Statistical calculations were performed using a statistical analysis package (SAS, version 9.3; SAS Institute; Cary, NC, USA). One-way analysis of variance (ANOVA) was performed for determination of the significance of the difference at the baseline of each constitution. Tukey's HSD test was used as a post hoc analysis. For the ANOVA method, the data were considered that there were four basic assumptions: the expected values of the errors are zero, the variances of all errors are equal to each other, the errors are independent, and they are normally distributed. Tests for homogeneity and normality were performed (see Supplementary Tables 1 and 2 in Supplementary Material available at http://dx.doi.org/10.1155/2013/171643). Differences were considered significant with *P* < 0.05.

### 2.4. Determination of the Sasang Constitution

The subjects' SC types were determined by the unique Sasang constitution diagnosis system (SCDS) developed by Korea Institute of Oriental Medicine (KIOM). In brief, the SCDS is a web-based SC analytical software which integrates four parts of an individual's characteristic information including anthropometric measures of body shape (10 points), facial contour analysis (10 points), voice features (5 points), and physiologic symptoms collected by a questionnaire (10 points). Once individual levels of all required items were entered into the SCDS system, it presented the percentage of each person's potential to be categorized as a SC type. Then, a person's SC type with the highest percentage is adopted as the person's final type [1] (Supplementary Table 3). Although there are four constitutions in Sasang typology, Tae-Yang (TY), Tae-Eum (TE), So-Yang (SY), and So-Eum (SE) types, the TY type was very rare in recruited subjects as in other studies; thus, we could not consider the TY type in this study.

### 2.5. Pyrosequencing

Metagenomic DNA was extracted from fecal samples using a FastDNA SPIN extraction kit (MP Biomedicals, Santa Ana, CA, USA), and the 16S rRNA gene (V1−V3 regions) was amplified from extracted DNA. Amplifications were performed as in previous reports using a barcoded fusion primer [22, 23] (http://oklbb.ezbiocloud.net/content/1001), using a C1000 Touch thermal cycler (Bio-Rad, Hercules, CA, USA). The amplified products were confirmed by 2% agarose gel electrophoresis and visualized using the Gel Doc system (Bio-Rad). Amplicons were purified using a QIAquick PCR purification kit (Qiagen, Valencia, CA, USA) and quantified using a PicoGreen dsDNA Assay kit (Invitrogen, Carlsbad, CA, USA). Equimolar concentrations of each amplicon from different samples were pooled and purified using an AMPure bead kit (Agencourt Bioscience, Beverly, MA, USA) and then amplified on sequencing beads by emulsion PCR. Sequencing reactions were performed using a Roche/454 GS Junior system according to the manufacturer's instructions.

### 2.6. Sequence Data Analysis

The analysis of pyrosequence data was conducted according to previous descriptions [23, 24]. Briefly, raw data for each sample were sorted by a unique barcode in the demultiplexing step, and low quality reads (average quality score <25 or read length <300 bp) were removed for further analysis. Pairwise sequence alignment and the hmm-search program of the HMMER 3.0 package [25] were used for trimming of primer sequences based on the profile of the 16S rRNA V1−V3 regions. For correction of sequencing errors, representative sequences in clusters of trimmed sequences were selected and considered for taxonomy identification. Individual reads were assigned their taxonomic positions according to the highest pairwise similarity among the top five BLASTN hits against the EzTaxon-e database [26]. Chimera sequences were removed by UCHIME [27]. The read numbers in each sample were normalized by random subsampling, and the diversity indices were calculated using the Mothur program [28]. Pyrosequencing reads obtained from this study are available in the EMBL SRA database under study number ERP002551 (http://www.ebi.ac.uk/ena/data/view/ERP002551).

## 3. Results and Discussion 

### 3.1. General Characteristics of Subjects

Sasang constitution of 40 subjects was determined by the SCDS, and characteristics and clinical markers were compared among SC groups (Table 1). SE, SY, and TE accounted for 32.5%, 32.5%, and 35%, respectively, of the total subjects. Expectedly, the TY type was not included in our study. Individuals having the TY type have been reported to be rare in the south part of Korean peninsula. Ninety-two percent of SY group (*n* = 13) were female, and the average age of this group (42.6 years old) was higher than that of other SC groups (SE: 32.9 versus TE: 29.1 years old) (*P* < 0.05). The height of SY group was lower than that of other groups, which may be partly due to the higher ratio of female and older ages in this group. Systolic and diastolic blood pressure as well as fasting blood sugar were not significantly different across groups. Significant differences were observed for all of the anthropometric and body composition associated variables: height, weight, waist circumference, BMI, fat percent, lean body mass, and fat percentage (*P* < 0.05). The height (159.8 cm) and lean body mass (21.3 kg) of SY group were significantly lower than those of the other groups (*P* < 0.001). The weight (67.5 kg), BMI (22.7), and waist circumference (850.1 mm) of TE group were significantly higher compared with the other groups (*P* < 0.001). Because a higher level of these parameters is often associated as risk factor of abdominal obesity, subjects in the TE group could be most vulnerable to obesity and metabolic syndrome [29]. The waist-hip ratio of SE group was significantly lower than that of the other groups (*P* < 0.05). Fat mass was the only variable that all three groups were significantly different: SE < SY < TE. Fat percentage of SE group was lower than that of the other groups (*P* < 0.001), which may be attributed to the higher male ratio (69.2%) in the SE group. Fasting blood sugar, triglyceride, HDL-cholesterol, and total cholesterol were not significantly different among the groups.

### 3.2. Identification of Gut Microbiota in Subjects

A total of 99,622 sequences were obtained and analyzed from fecal samples of 40 subjects, which consisted of 14 Tae-Eum, 13 So-Yang, and 13 So-Eum constitutions; 32,518 sequences (452.9 ± 9.6 bp) obtained from TE subjects, 30,048 sequences (453.4 ± 9.3 bp) from SY, and 37,056 sequences (454.2 ± 7.1 bp) from SE were analyzed for comparison of gut microbiota. Calculated diversity indices after normalization of read sizes in each sample were compared (Table 2). The read numbers of TE_11, 12, 13, 14, SY_1, 5, 13, and SE_8, 9 samples were less than those of normalized reads. However, the number of observed OTUs and Chao 1 was not influenced by a lower read number. For example, the number of observed OTUs of TE_13 was 463 by 1,677 total reads, while that of the observed OTUs of TE_9 was 138 by 2,000 normalized reads. This indicated that the statistical analyses of samples were not affected by read number of samples. The values of Good's coverage exceeded 0.77 in all samples. Estimated values in Table 2 varied within constitution type. Although averages of Shannon indices of three constitutions were similar, ranging from 3.66 to 3.87, the rarefaction curves showed the difference of gut microbiota among constitutions (Supplementary Figure 1). The richness of bacterial communities obtained from TE constitution was relatively higher than that of SY and SE. The slopes of rarefaction curves from bacterial communities of SY and SE varied and those of SE varied more than those of SY, which could indicate the difference of gut microbiota in three constitutions.

**Figure 1:**
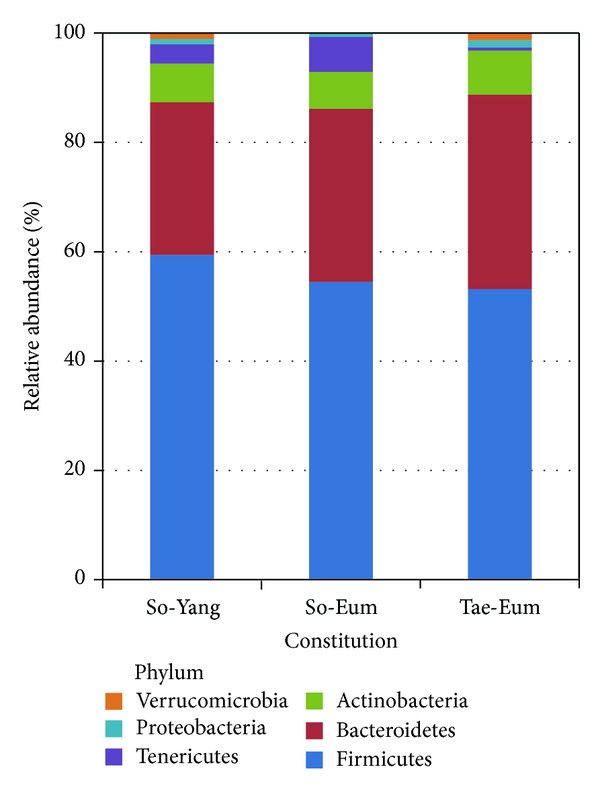
The average compositions of bacterial communities obtained from three different constitutions (So-Yang, So-Eum, and Tae-Eum) were compared at the phylum level. The phylum represented by each color is defined at the right of the figure.

The average phyla compositions of bacterial communities from three constitutions were analyzed and compared (Figure 1). The average compositions of phyla were similar among constitutions; two phyla of Firmicutes (ranging from 53.2% to 59.4% of total reads) and Bacteroidetes (from 27.9% to 35.6%) predominated, followed by phylum of Actinobacteria (ranging from 6.8% to 8.0%). The proportion of Tenericutes was more than 3.6% in samples of SY and SE, whereas it is under 0.6% in samples of TE. The proportion of Verrucomicrobia in SE was (0.1%) lower than those of SY (1.0%) and TE (1.3%). Although the proportion of each phylum varied, the average compositions of predominant bacteria from three constitutions were similar. This could be because all of the tested subjects were healthy persons and the gut microbiota play a normal role in their body. However, the compositions of bacterial communities from individuals varied within constitutions (Supplementary Figure 2). These variations reflected the individual differences of gut microbiota.

**Figure 2:**
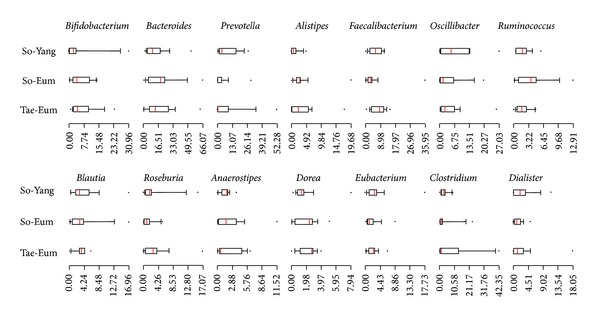
The median plots of genera from three constitutions were compared. Bar scale indicates the proportion of each genus. The median value of the genus proportion in one of each constitution which was more than 1% of analyzed reads was selected for comparison.

The variations of bacterial communities were compared among samples using the median plots of genus proportions (Figure 2). Selected genera, which have over 1% proportion of median value in one of each group, were compared among SC groups. The proportions of *Prevotella*, *Oscillibacter*, and *Dialister* were higher in SY than in SE and TE. The relative abundances of *Bacteroides* and *Ruminococcus* were higher in SE, compared with the other two constitutions, while those of *Faecalibacterium* and *Roseburia* were higher in TE. Median values of *Bifidobacterium*, *Oscillibacter*, *Dorea*, and *Dialister* in samples of SE were similar to those of TE. Although differences among constitutions were observed in median plots of genus, various compositions of bacterial communities were investigated within each constitution (Supplementary Figure 2). These individual variations caused difficulty in analysis and comparison of gut microbiota among SC groups.

### 3.3. Variations of Gut Microbiota according to Gender and Age

**Figure 3:**
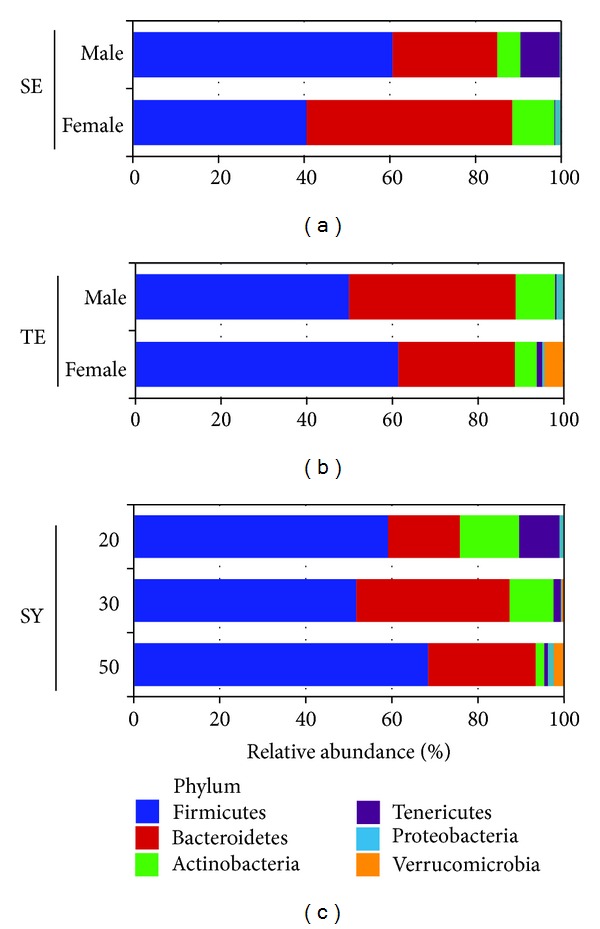
Comparison of average bacterial community obtained from different genders and ages at the phylum level. Bacterial communities from So-Eum and Tae-Eum were compared between male and female, due to an insufficient number of males in So-Yang subjects. Samples of So-Yang constitution had relatively various age ranges; thus, comparison of different ages was performed within So-Yang. The range of ages was divided into groups of 20 (range from 20 to 29 years old), 30 (30–39), and 50 (50–59).

Variations in gut microbial communities obtained from healthy persons have been reported in several microbiome studies [11, 12, 30]. Driven factors for variations in human microbiome were studied and complex factors including diets, drugs, and host disease influence gut microbiota. Among various factors, gender and age are also critical factors to variations of gut microbiota. Differences of gut microbial communities were related to gender in macaques and human [31, 32], and interindividual variations were influenced by age [33, 34]. Therefore, we analyzed the differences of bacterial communities within each constitution according to gender and age. Comparisons of bacterial communities between male and female were performed in SE (nine males and four females) and TE (ten males and four females) samples (Figure 3). The average compositions of bacterial communities between male and female samples were compared at the phylum level. The proportion of each phylum was different in male and female samples of both constitutions. The proportion of Firmicutes was higher in male samples (60.7%) of SE than in female samples (40.6%), whereas that of Bacteroidetes was higher in female samples (48.1%) than in male samples (24.4%). The proportion of Tenericutes was 9.2% in male samples and 0.2% in female samples. In contrast to SE, the proportion of Firmicutes was higher in female samples (61.4%) of TE than in male samples (49.9%), while that of Bacteroidetes was higher in male samples (38.9%) than in female samples (27.2%). Of particular interest, the phylum of Verrucomicrobia was investigated only in female samples of TE. SY subjects consisted of 12 females and one male; thus, comparison between male and female was not possible in this constitution. Age-related comparisons were performed in SY samples, because most samples of SE and TE consisted of age ranging from 20 to 29 years old. The range of age in SY samples was divided into groups of 20 (range from 20 to 29 years old), 30 (30–39), and 50 (50–59). Age-related differences in bacterial community are shown in Figure 3. Increased Firmicutes was observed in group of 50, while Actinobacteria and Tenericutes were decreased with aging. Gut microbiota could be influenced by aging due to dietary habit and altered physical functions of the body [33]. Gut microbiota appear to become more stable throughout adulthood, although some studies have reported a higher abundance of *Bifidobacteria* and *Clostridia* in adolescents, compared with adults [35]. Although this result was generated from a small number of subjects, the differences between gender and age were clearly observed in comparison. These differences indicated that age and gender were considerable factors for individual variations of gut microbiota.

### 3.4. Comparison of Gut Microbiota between SE and TE

**Figure 4:**
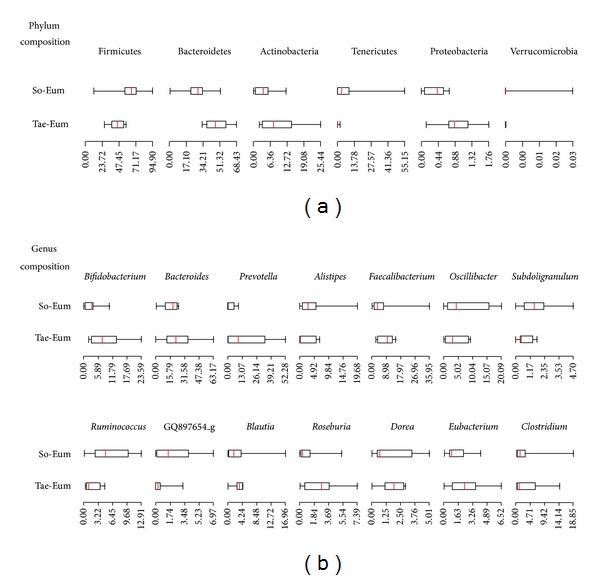
Bacterial communities obtained from the 20-year male group of So-Eum and Tae-Eum were compared using median plots. (a) Differences of phyla composition were compared between two constitutions. (b) Detailed differences of genera composition were analyzed in two constitutions. The identified genus name of GQ897654 indicates that these sequences have the highest similarity to uncultured bacteria GQ897654 (GenBank accession number).

For comparison of bacterial communities in the gut among constitutions without considerable variation factors, male samples in the 20-year age group (range from 20 to 29 years old) in SE (*n* = 8) and TE (*n* = 8) were selected and analyzed using median plots (Figure 4). Differences of gut microbiota between SE and TE became clearer when age and gender were adjusted for the analysis. The relative abundance of Firmicutes in SE (61.7% of median value) samples was higher than that in TE (42.1%), whereas the proportions of Bacteroidetes (44.4%) and Actinobacteria (5.6%) were higher in TE than in SE (29.2% of Bacteroidetes and 3.7% of Actinobacteria) samples. Detailed differences were observed at the genus level. Proportions of *Bifidobacterium* within Actinobacteria were higher in TE, compared with SE samples. Ratios of *Bacteroides* within Bacteroidetes showed similar median values in both constitutions, while the relative abundance of *Prevotella* and *Alistipes* was different in SE and TE samples. The median values of *Faecalibacterium* and *Ruminococcus* within Firmicutes were the most different genera between two constitutions. Other genera within Firmicutes are shown in Figure 4.

**Figure 5:**
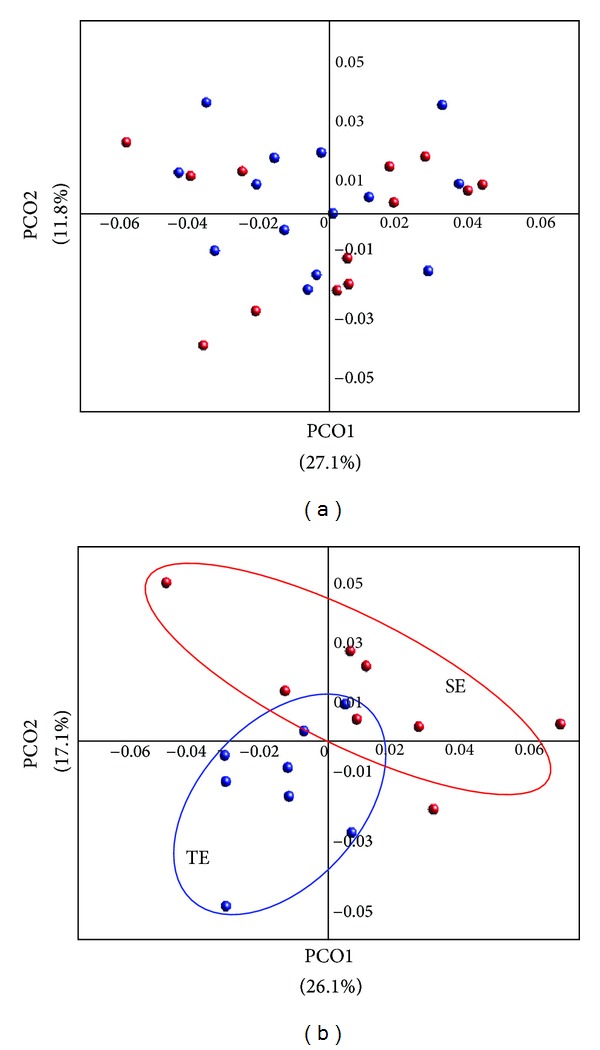
Similarities between bacterial communities originating from So-Eum and Tae-Eum were compared using principal coordinates analysis (PCoA). Similarities were calculated by the Fast UniFract. (a) All communities obtained from So-Eum and Tae-Eum, were compared. (b) Communities of males, 20-year old groups from two constitutions were compared in PCoA plots. Red circle indicates the community of So-Eum and blue circle indicates those of Tae-Eum. The percent variation is explained by each PCoA axis in axis labels.

The difference of bacterial communities between SE and TE was also investigated in PCoA plots based on UniFrac distance (Figure 5). The communities of SE and TE were distributed and mixed without consideration for gender and age (Figure 5(a)). This result was consistent with difference of average bacterial community (Figure 2 and Supplementary Figure 2). Bacterial communities originating from male samples of similar age (ranging from 20 to 29 years old) in SE and TE groups showed distinguished pattern according to their constitution (Figure 5(b)), consistent with the median plot analysis shown in Figure 4. This result indicates that gender and age were considerable factors in comparison of gut bacterial communities between SC groups. In general, SE constitution was known to have a tendency of dyspepsia and diarrhea due to a weak gastrointestinal system and “cold” nature, whereas few gastrointestinal problems are associated with TE constitution due to the relatively strong digestion and adsorption function in traditional medicine [36]. These differences could be related to the different microbial communities in the gut and their different functions.

## 4. Conclusion

In this study, we analyzed the gut microbiota in three SC types (SY, SE, and TE) using 16S rRNA gene-based pyrosequencing as a pilot study. Individual variations of gut microbiota in each SC as well as gut microbial variation across SC were investigated. Firmicutes and Bacteroidetes were the common, predominant phyla followed by Actinobacteria in all SC types. The median plot analysis suggested that Firmicutes and Bacteroidetes may be more abundant in SE and TE, respectively, in the male subjects of 20–29 years old. At the genus level, *Bifidobacterium* and *Bacteroides* manifested the difference between SE and TE types. For anthropometry, body weight, body mass index, and waist circumference of the TE type were significantly higher than those of the other types. Although we could not confirm the associations between gut microbial variation with SC types in conjunction with age and gender thoroughly, the gut microbiota could be used one of the scientific factors in deciding SC type in traditional Korean medicine. Findings in this study should be investigated in depth with a larger sample size to increase our understanding for the association between gut microbial variation and SC types.

## Supplementary Material

Supplementary Table 1: P-values for Homogeneity.
Supplementary Table 2: P-values for Normality Test.
Supplementary Table 3: Points in KIOM constitution analysis system.
Supplementary Figure 1: The rarefaction curves of three different constitutions were obtained after normalization of read size in each sample (Table 2). TE indicates Tae-Eum, SY indicates So-Yang, and SE indicates So-Eum.
Supplementary Figure 2: The compositions of bacterial communities originating from each sample of Tae-Eum are presented in double pie charts. The inner circle indicates the composition of the phylum, and the outer circle indicates the composition of the genus. Each color is defined below the figure. The nomenclatures of phylotypes are based on the EzTaxon-e database [25].
Supplementary Figure 3: The double pie chart of bacterial communities obtained from each sample of So-Yang. The inner circle indicates the composition of the phylum, and the outer circle indicates the composition of the genus.
Supplementary Figure 4: The composition of bacterial communities obtained from each sample of So-Eum in the double pie chart. The inner circle indicates the composition of the phylum, and the outer circle indicates the composition of the genus.

## Figures and Tables

**Table 1:** Characteristics of subjects in each constitution.

Characteristics	So-Yang (SY)	So-Eum (SE)	Tae-Eum (TE)	*P* value^a^	Comparison significance^b^
Sex (M/F)	1/12	9/4	8/6	0.0036	
Age (year)	42.6 ± 14.7	29.1 ± 9.1	32.9 ± 14.8	0.0356	SY*≠*SE
Systolic (mmHg)	110.4 ± 12.6	113.3 ± 8.4	114.8 ± 10.9	0.5670	
Diastolic (mmHg)	70.0 ± 11.1	69.0 ± 9.0	70.6 ± 11.2	0.9201	
Heart rate	76.7 ± 11.0	71.0 ± 6.8	73.0 ± 4.4	0.1849	
Height (cm)	159.8 ± 3.3	170.3 ± 7.7	172.2 ± 8.0	<0.0001	TE*≠*SY, SE*≠*SY
Weight (kg)	53.7 ± 4.0	58.9 ± 7.9	67.5 ± 7.3	<0.0001	TE*≠*SE, TE*≠*SY
BMI (kg/m^2^)	21.0 ± 1.2	20.2 ± 1.3	22.7 ± 1.0	<0.0001	TE*≠*SE, TE*≠*SY
Waist circumference (mm)	768.0 ± 72.3	745.1 ± 58.0	850.1 ± 51.4	0.0002	TE*≠*SE, TE*≠*SY
Waist-hip ratio	0.852 ± 0.1	0.826 ± 0.0	0.867 ± 0.0	0.0338	TE*≠*SE, SY*≠*SE
Lean body mass (kg)	21.3 ± 2.8	26.8 ± 4.1	28.0 ± 5.0	0.0004	TE*≠*SY, SE*≠*SY
Fat mass (kg)	14.4 ± 3.1	10.6 ± 2.8	17.1 ± 2.6	<0.0001	SY*≠*SE*≠*TE
Fat percentage	26.8 ± 5.4	18.0 ± 3.9	25.7 ± 5.2	<0.001	SY*≠*SE, TE*≠*SE
Fasting blood sugar (mg/dL)	102.8 ± 13.3	101.8 ± 10.9	99.1 ± 12.8	0.7171	
Triglyceride (mg/dL)	106.2 ± 86.0	86.4 ± 41.5	94.6 ± 37.3	0.6895	
HDL-cholesterol (mg/dL)	61.5 ± 13.1	60.9 ± 14.5	54.7 ± 12.6	0.3499	
Total cholesterol (mg/dL)	185.4 ± 30.5	164.8 ± 29.5	180.0 ± 29.4	0.2003	

^a^Values derived from ANOVA test, except for the variable “Sex” which was from the chi-square test.

^b^Results obtained from posthoc analysis Tukey's HSD test.

**Table 2:** Summary of statistical calculations obtained from pyrosequences.

	Total reads	Normalized reads	Average length (bp)	Observed OTUs	Estimated OTUs (Chao1)	Shannon index	Good's coverage
SY_1	1,511	1,511	457.8	427	2,144.06	4.64	0.78
SY_2	2,296	2,000	453.5	327	1,933.55	3.76	0.87
SY_3	2,552	2,000	456.5	404	2,399.63	4.05	0.85
SY_4	2,701	2,000	476.0	177	453.47	3.16	0.95
SY_5	1,669	1,669	451.2	298	1,320.13	3.80	0.87
SY_6	2,322	2,000	442.6	377	2,041.64	3.94	0.86
SY_7	2,418	2,000	460.0	314	1,519.40	2.92	0.88
SY_8	2,109	2,000	440.5	488	3,114.00	4.08	0.80
SY_9	2,257	2,000	443.3	490	2,762.50	4.14	0.80
SY_10	2,630	2,000	458.4	376	2,313.00	3.66	0.85
SY_11	2,264	2,000	455.4	362	2,121.57	3.83	0.86
SY_12	3,336	2,000	451.4	301	1,664.05	3.45	0.88
SY_13	1,983	1,983	447.4	586	2,615.50	4.93	0.77
SE_1	2,326	2,000	451.5	414	2,638.44	4.23	0.83
SE_2	3,858	2,000	463.3	182	1,342.00	2.02	0.93
SE_3	2,896	2,000	445.4	318	1,824.95	3.61	0.87
SE_4	2,689	2,000	452.2	334	1,569.04	3.93	0.88
SE_5	2,167	2,000	455.9	397	1,953.90	4.07	0.85
SE_6	6,180	2,000	471.6	109	1,110.25	0.93	0.96
SE_7	2,509	2,000	453.6	343	1,688.38	3.38	0.87
SE_8	1,933	1,933	455.6	512	3,604.28	4.71	0.78
SE_9	1,777	1,777	454.8	471	2,863.34	4.70	0.79
SE_10	2,084	2,000	452.4	525	3,622.71	4.87	0.79
SE_11	3,903	2,000	451.6	268	1,746.31	2.81	0.89
SE_12	2,345	2,000	443.6	460	2,509.87	4.11	0.82
SE_13	2,389	2,000	453.3	455	1,641.52	4.24	0.83
TE_1	2,065	2,000	453.2	439	2,342.10	4.18	0.83
TE_2	2,999	2,000	438.7	392	2,439.54	3.36	0.84
TE_3	2,420	2,000	451.8	449	3,091.64	4.29	0.82
TE_4	2,564	2,000	447.1	329	2,128.53	3.22	0.87
TE_5	2,659	2,000	446.4	411	3,125.25	4.23	0.84
TE_6	2,303	2,000	454.5	433	4,909.94	3.54	0.81
TE_7	2,245	2,000	451.6	354	2,586.56	3.86	0.86
TE_8	2,378	2,000	452.8	411	2,511.13	4.29	0.84
TE_9	4,019	2,000	478.4	138	478.09	2.91	0.96
TE_10	2,114	2,000	449.7	464	2,849.54	4.48	0.82
TE_11	1,360	1,360	467.8	93	212.25	2.78	0.96
TE_12	1,955	1,955	447.5	424	2,318.69	4.08	0.83
TE_13	1,677	1,677	451.6	463	2,619.44	4.53	0.78
TE_14	1,760	1,760	449.8	440	2,825.54	4.01	0.79
