# Supplementary material for: Comparison of Gut Microbiota between Sasang Constitutions
Source: Evid Based Complement Alternat Med. 2013 Dec 25;2013:171643. doi: 10.1155/2013/171643 (PMC3886231; doi:10.1155/2013/171643)
Supplement: Supplementary file 1 — Supplementary Table 1: P-values for Homogeneity. Supplementary Table 2: P-values for Normality Test. Supplementary Table 3: Points in KIOM constitution analysis system. Supplementary Figure 1: The rarefaction curves of three different constitutions were obtained after normalization of read size in each sample (Table 2). TE indicates Tae-Eum, SY indicates So-Yang, and SE indicates So-Eum. Supplementary Figure 2: The compositions of bacterial communities originating from each sample of Tae-Eum are presented in double pie charts. The inner circle indicates the composition of the phylum, and the outer circle indicates the composition of the genus. Each color is defined below the figure. The nomenclatures of phylotypes are based on the EzTaxon-e database [25]. Supplementary Figure 3: The double pie chart of bacterial communities obtained from each sample of So-Yang. The inner circle indicates the composition of the phylum, and the outer circle indicates the composition of the genus. Supplementary Figure 4: The composition of bacterial communities obtained from each sample of So-Eum in the double pie chart. The inner circle indicates the composition of the phylum, and the outer circle indicates the composition of the genus. [file 171643.f1.pdf]

Supplementary data

Supplementary table 1. P-values for Homogeneity

Characteristics	p-value
Age (year)	0.2130
Systolic (mmHg)	0.2662
Diastolic (mmHg)	0.5789
Heart rate	0.0856
Height (cm)	0.0708
Weight (kg)	0.0654
BMI (kg/m2)	0.7684
Waist circumference (mm)	0.5403
Waist-hip ratio	0.0293
Lean body mass (kg)	0.1028
Fat mass (kg)	0.8744
Fat percent	0.6345
Fasting blood sugar (mg/dl)	0.7728
Triglyceride (mg/dl)	0.3707
HDL-cholesterol (mg/dl)	0.8252
Total cholesterol (mg/dl)	0.9808

p-value derived from Levene’s test

Supplementary table 2. P-values for Normality Test

Characteristics	So-Yang (SY)	So-Eum (SE)	Tae-Eum (TE)	p-value[a]	p-value[b]
Age (year)	0.1923	0.0018	0.0012	0.0356	0.1003
Systolic (mmHg)	0.2945	0.9336	0.8553	0.5670	0.4724
Diastolic (mmHg)	0.3204	0.1404	0.4454	0.9201	0.9015
Heart rate	0.0510	0.5544	0.3632	0.1849	0.6062
Height (cm)	0.7463	0.3389	0.3122	<0.0001	0.0008
Weight (kg)	0.9127	0.7658	0.0667	<0.0001	0.0003
BMI (kg/m2)	0.6166	0.7728	0.0426	<0.0001	<.0001
Waist circumference (mm)	0.2115	0.5582	0.0320	0.0002	0.0004
Waist-hip ratio	0.3254	0.0574	0.1529	0.0338	0.0167
Lean body mass (kg)	0.1213	0.2806	0.0355	0.0004	0.0013
Fat mass (kg)	0.0765	0.2415	0.9543	<0.0001	<.0001
Fat percent	0.0320	0.9969	0.4035	<0.001	0.0002
Fasting blood sugar (mg/dl)	0.8908	0.7781	0.0376	0.7171	0.5589
Triglyceride (mg/dl)	<.0001	0.0039	0.26253	0.6895	0.8048
HDL-cholesterol (mg/dl)	0.0500	0.1080	0.0625	0.3499	0.3317
Total cholesterol (mg/dl)	0.9621	0.6964	0.6128	0.2003	0.2112

[a] p-value derived from one-way ANOVA
[b] p-value derived from Kruskal-Wallis test

Supplementary table 3. Points in KIOM constitution analysis system

Constitution	Points	Constitution	Points	Constitution	Points
TE_1	42	SY_1	61	SE_1	61
TE_2	66	SY_2	42	SE_2	42
TE_3	61	SY_3	66.3	SE_3	79
TE_4	79	SY_4	42	SE_4	93
TE_5	61	SY_5	42	SE_5	74
TE_6	74	SY_6	42	SE_6	61
TE_7	73	SY_7	61	SE_7	66
TE_8	42	SY_8	42	SE_8	66
TE_9	42	SY_9	42	SE_9	74
TE_10	61	SY_10	42	SE_10	40
TE_11	42	SY_11	42	SE_11	61
TE_12	40	SY_12	42	SE_12	42
TE_13	66	SY_13	61	SE_13	61.4
TE_14	79				
Mean (SD)	59.1(14.8)[ab]		48.3(9.9)[a]		63.1(15.4)[b]
