# Supplementary figures and images for: Comparison of Gut Microbiota between Sasang Constitutions
Source: Evid Based Complement Alternat Med. 2013 Dec 25;2013:171643. doi: 10.1155/2013/171643 (PMC3886231; doi:10.1155/2013/171643)

Supplementary figure 1

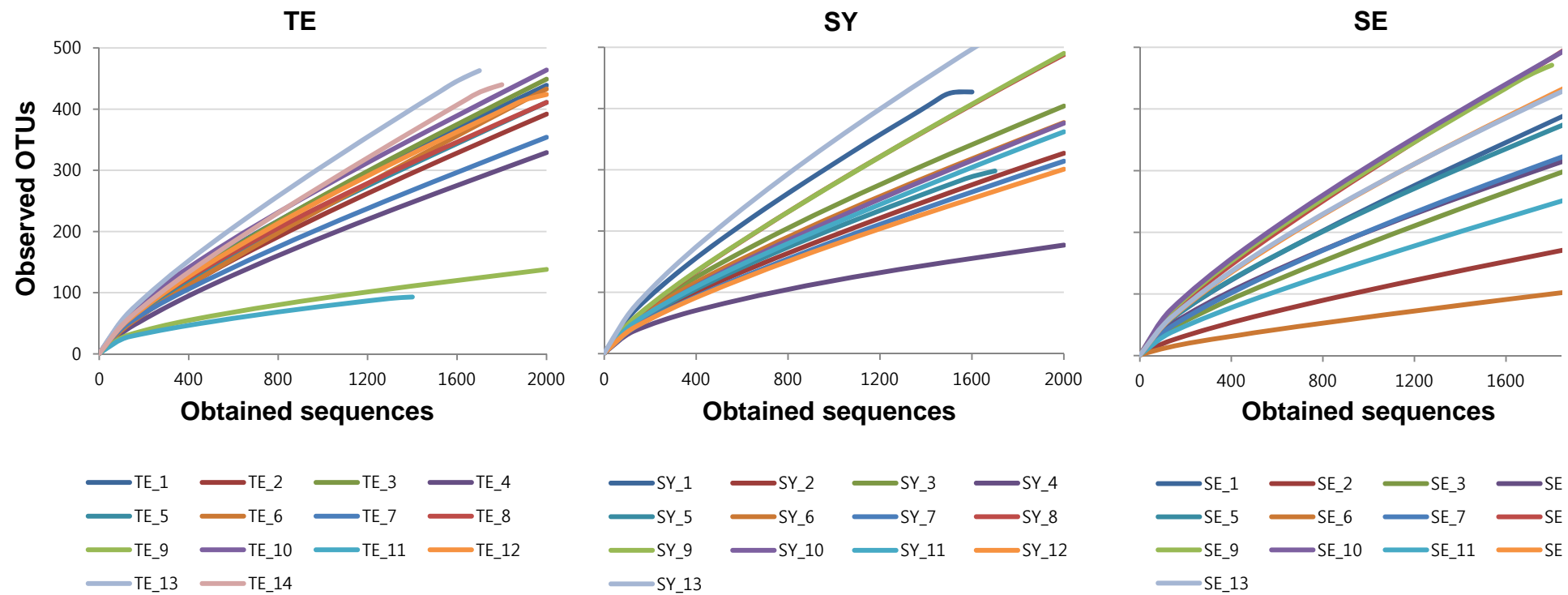

Supplementary figure 2

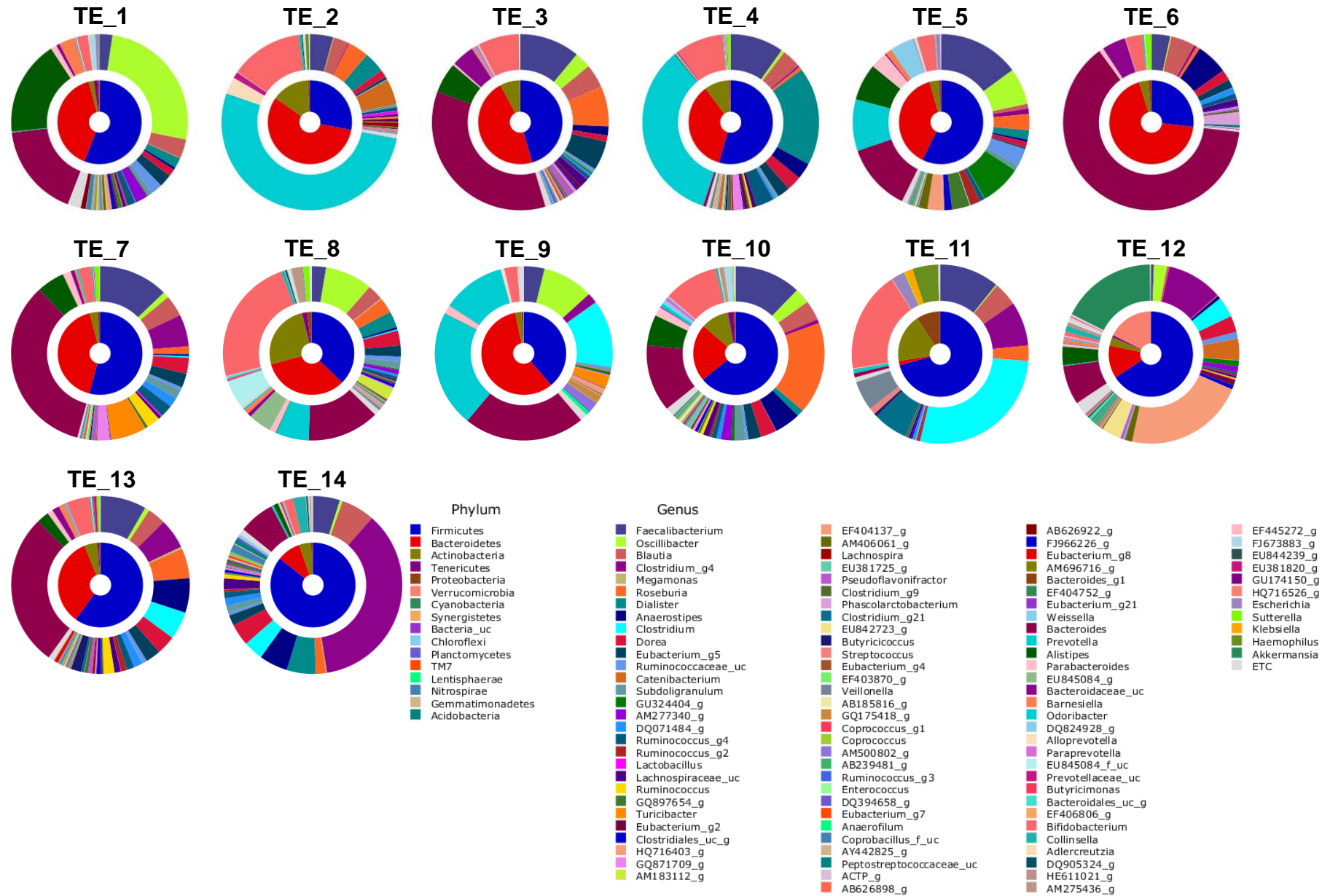

Supplementary figure 3

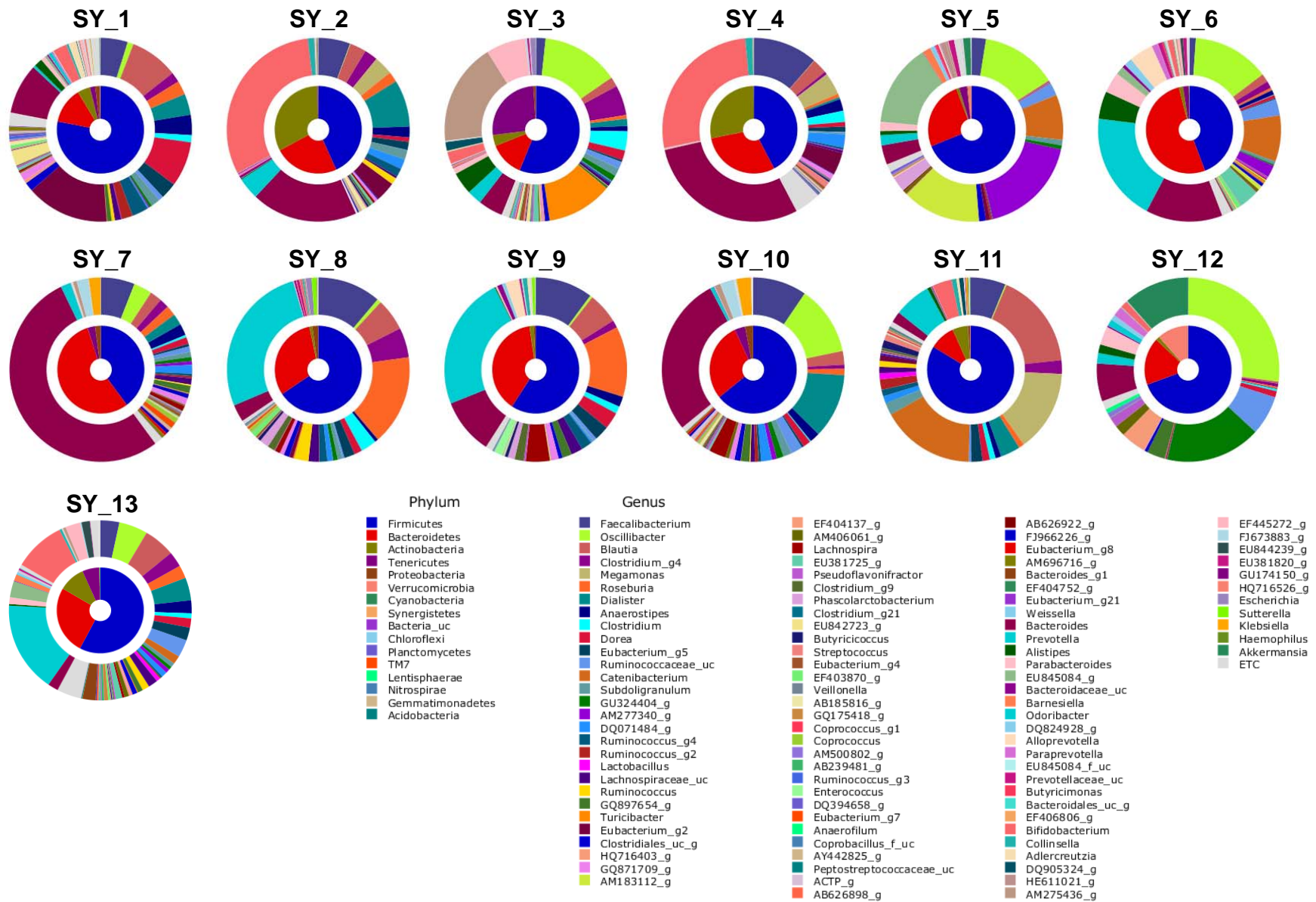

Supplementary figure 4

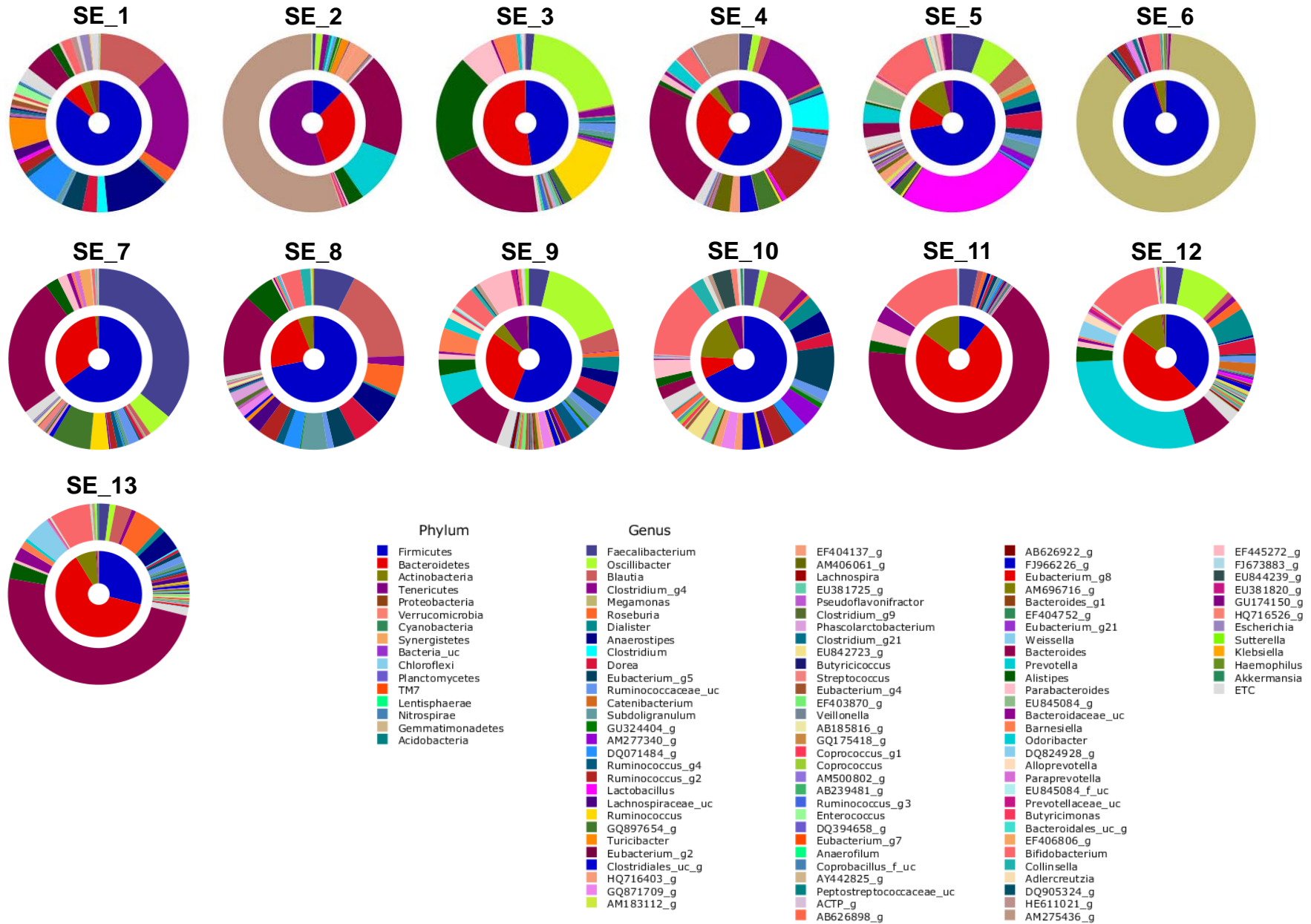

Supplement: Supplementary file 2 [file 171643.f2.pdf]
